# Screening for antibacterial activity of some Turkish plants against fish pathogens: a possible alternative in the treatment of bacterial infections

**DOI:** 10.1080/13102818.2015.1006445

**Published:** 2015-02-17

**Authors:** Hakan Turker, Arzu Birinci Yıldırım

**Affiliations:** ^a^ Biology Department, Science Faculty, Abant Izzet Baysal University, Bolu, Turkey; ^b^ Field Crops Department, Agricultural and Natural Sciences Faculty, Abant Izzet Baysal University, Bolu, Turkey

**Keywords:** Turkish plants, fish bacteria, ethanol extract, water extract, antibacterial activity

## Abstract

The antibacterial activity of ethanolic and aqueous crude extracts from 36 plants in Turkey, including seven endemic species, against fish pathogens was studied using the disc diffusion assay. The extract that was most active against all microbial strains, except *Aeromonas salmonicida*, was that of *Dorycnium pentaphyllum*. Some of the extracts also showed a very broad spectrum of potent antimicrobial activity. The extract of *Anemone nemorosa* showed the highest antimicrobial activity against *Vibrio anguillarum*. *V. anguillarum*, a Gram-negative bacterium, appeared to be the most susceptible to the plant extracts used in this experiment. To the best of our knowledge, this is the first report on the antimicrobial activity of 11 of the studied plants. The preliminary screening assay indicated that some of the Turkish plants with antibacterial properties may offer alternative therapeutic agents against bacterial infections in aquaculture industry.

## Introduction

*The State of World Fisheries and Aquaculture* reveals that the sector produced a record about 122 million tonnes of fish for human food, providing more than 4.3 billion people with about 15% of their animal protein intake.[1,2] Of this supply, almost half is derived from aquaculture production. Although aquaculture has grown more rapidly than all other animal food production sectors, it is impeded by unpredictable mortality due to negative interactions with high stocking rates and diseases caused by pathogenic bacteria.

In modern large-scale fish farms, a wide variety of disinfectants and antibiotics are given preference as agents used against pathogens. However, the environmentally friendly and user-safe chemicals can be used as an alternative to replace these agents. For example, malachite green has been banned in the European Union and the USA.[3] The compounds with antibiotic activity are designed to inhibit the growth and kill pathogenic bacteria in aquaculture production. Romero et al. [4] indicated that the usage of antimicrobial agents in aquaculture causes the development and spread of antibacterial-resistant bacteria, resistance genes and the presence of antimicrobial residues in aquaculture products and the environment.[4]

The global concerns about bacterial antibiotic resistance and antibiotic residues have increased. Recently, the US Centers for Disease Control and Prevention published a report of the threat that the country faces from the antibiotic-resistant organisms.[5] Therefore, there is a need for development of alternative therapies against bacterial pathogens in aquaculture production. Several alternatives to the usage of antibiotics have been applied successfully in aquaculture.[4] One such alternative is the use of probiotics to avoid bacterial infection in aquacultures.[6,7] Other sources of alternative treatment are essential oils [8,9] and plant extracts,[10–14] which have been used *in vivo* as antibacterial agents to control bacterial infections. These compounds may constitute alternative prophylactic and therapeutic agents in aquaculture because of their antibacterial properties.

The use of plant extracts and other alternative forms of medical treatment against pathogens is gaining great popularity based on scientific interest and public awareness.[15] Traditional medicine has been used in some communities from Central Asia to Anatolia for thousands of years. Herbal treatments are currently the most popular form of traditional medicine, generating billions of dollars in revenue, and are highly lucrative in the international marketplace.[15,16] In some Asian, African and many developed countries, 70% to 80% of the population depends on traditional medicine for primary healthcare and uses some form of alternative or complementary medicine.[17–21]

Turkey has rich plant diversity and the endemism ratio is relatively high when compared with other European countries.[22–24] In Turkey, 8988 native plant species have been described with 2991 endemic plant species and the endemism ratio is about 33.3%.[13,24,25] In this rich variety of plant species, their potential antibacterial activity needs to be explored and scientifically verified. Therefore, the aim of this study was to evaluate the antibacterial activity of alcoholic and aqueous extracts obtained from 36 Turkish plants on most frequently isolated bacteria in aquaculture industry.

## Materials and methods

### Preparation of plant extracts

Plants were collected from the region of Bolu (Turkey) and were identified according to [26]. The original specimens were deposited at the Abant Izzet Baysal University Herbarium, Bolu. All plant samples and collection numbers are listed in Table 1. All collected plants were oven dried at 40 °C and water and ethanol (EtOH) extracts were obtained. For aqueous extraction, 20 g from each powdered plant sample were extracted with 200 mL of water at 80 °C in a water bath for 12 h and then filtered. Water was evaporated using a lyophilizator. For alcoholic extractions, 20 g of plant sample were Soxhlet extracted with 350 mL of EtOH at 60 °C for 12 h and the liquid portion was evaporated under vacuum. For antibacterial assay, each extract was dissolved in sterile distilled water in order to obtain a final concentration of 100 mg/mL. Plant materials, designation of treatments and yield (%) for each extraction are summarized in Table 1.

**Table 1.** List of the studied plant species, accession numbers, plant parts and extract yields.

Family and plants species	Collection number	Part used	Yield (%)*
Rosaceae			
*Crataegus monogyna* Jacq.	AUT-2035	Fresh fruits	0.6
			4.1
*Pyracantha coccinea* Roemer	AUT-2032	Fresh fruits	2.9
			4.1
*Fragaria vesca* L.	AUT-2037	Fresh fruits	7.0
			7.1
*Rubus caesius* L.	AUT-2033	Fresh fruits	2.7
			4.4
*Alchemilla mollis* (Buser) Rothm.	AUT-2019	Aerial	2.0
			1.2
Caprifoliaceae			
*Viburnum opulus* L.	AUT-2029	Fresh fruits	1.6
			4.2
*Viburnum lantana* L.	AUT-2030	Fresh fruits	1.8
			3.0
Apiaceae			
*Eryngium campestre* L.	AUT-2018	Aerial	1.7
var. *virens*			1.8
*Astrantia maxima* Pallas	AUT-1949-Endemic	Aerial	16.0
subsp. *haradjianii* (Grintz.) Rech. fil.			17.5
Lamiaceae			
*Mentha longifolia* (L.) Hudson	AUT-1937	Aerial	16.0
subsp. *typhoides* (Briq.) Harley var. *typhoides*			7.3
*Lamium crinitum* Montbret & Aucher ex Bentham	AUT-1945	Aerial	21.3
			12.0
*Phlomis russeliana* (Sims) Bentham	AUT-1946-Endemic	Aerial	6.24
			8.6
*Phlomis armeniaca* Willd.	AUT-1954-Endemic	Aerial	4.4
			8.9
*Sideritis taurica* Stephan	AUT-1953	Aerial	12.8
			15.4
Fabaceae			
*Dorycnium pentaphyllum* Scop.	AUT-2020	Aerial	2.2
subsp. *anatolicum* (Boiss.) Gams			5.6
*Coronilla varia* L.	AUT-2022	Aerial	3.3
subsp. v*aria*			7.0
*Onobrychis oxydonta* Boiss.	AUT-2026	Aerial	1.6
			2.4
*Astragalus brachypterus* Fischer	AUT-1947-Endemic	Aerial	8.6
			7.6
Asteraceae			
*Doronicum orientale* Hoffm.	AUT-2021	Aerial	4.1
			5.3
*Senecio castagneanus* DC.	AUT-1952-Endemic	Aerial	14.0
			15.0
*Centaurea triumfettii* All.	AUT-1940	Aerial	10.0
			13.3
*Ptilostemon afer* (Jacq.) Greuter	AUT-1948-Endemic	Aerial	14.7
subsp. *eburneus*			7.0
Scrophulariaceae			
*Rhinanthus angustifolius* C.C. Gmelin	AUT-2025	Aerial	2.5
			2.5
Boraginaceae			
*Cynoglossum montanum* L.	AUT-1943	Aerial	7.6
			14.1
*Echium orientale* L.	AUT-1950-Endemic	Aerial	8.2
			4.6
Liliaceae			
*Polygonatum orientale* Desf.	AUT-1942	Aerial	10.4
			6.3
*Fritillaria pontica* Wahlenb.	AUT-2023	Aerial	2.8
			1.9
Thymelaeaceae			
*Daphne oleoides* Schreber	AUT-1941	Leaves and flowers	3.3
subsp. *oleoides*			28.7
Ranunculaceae			
*Anemone nemorosa* L.	AUT-1955	Aerial	19.3
			29.0
Araceae			
*Arum euxinum* R. Mill	AUT-1951-Endemic	Aerial	9.7
			30.8
Solanaceae			
*Hyoscyamus niger* L.	AUT-1944	Aerial	15.4
			19.8
Aristolochiaceae			
*Asarum europaeum* L.	AUT-2024	Aerial	2.7
			1.6
Campanulaceae			
*Campanula glomerata* L.	AUT-2027	Aerial	3.0
subsp. *hispida* (Witasek) Hayek			3.0
*Campanula olympica* Boiss.	AUT-2028	Aerial	2.0
			2.9
Hypericaceae			
*Hypericum perforatum* L.	AUT-1938	Aerial	14.3
			18.7
*Hypericum linarioides* Bosse	AUT-1939	Aerial	10.3
			27.8

*Yield (%) = (weight of extract (g)/20 g of powdered plant sample) × 100.

### Fish bacteria

Four Gram-negative (*Aeromonas hydrophila*, *Aeromonas salmonicida*, *Vibrio anguillarum* and *Yersinia ruckeri*) and three Gram-positive bacteria (*Enterococcus faecalis*, *Lactococcus garvieae* and *Streptococcus agalactia*) were used in this study. *A. hydrophila* (ATCC 19570) and *S. agalactia* (Pasteur Institute 55118) were purchased from Refik Saydam Hygiene Center Culture Collection. *V. anguillarum*, *Y. ruckeri* and *L. garvieae* were provided by Dr Altınok, Sürmene Faculty of Marine Science, Karadeniz Technical University, Trabzon, Turkey; *E. faecalis* by Dr Koyuncu, Faculty of Fisheries, Mersin University, Mersin, Turkey and *A. salmonicida* by Dr Kırkan, Faculty of Veterinary Medicine, Adnan Menderes University, Aydın, Turkey.

### Antibacterial bioassay

The disc diffusion assay (Kirby–Bauer Method) was used to screen for antibacterial activity.[27] A pure culture of each bacterial strain was grown on tryptic soy agar (Acumedia®) and incubated at 25 °C for *A. salmonicida* and *Y. ruckeri*, and at 37 °C for the other bacteria for two days. The turbidity of each broth culture was adjusted with saline to obtain turbidity visually comparable to that of a 0.5 McFarland standard and then Mueller Hinton agar plates (Acumedia®) were inoculated by using cotton swabs. All extracts were sterilized by filtering through a 0.22 μm filter (Millex®) and sterile filter paper discs (Glass Microfibre filters, Whatman®; 6 mm in diameter) were impregnated with 13 µL of the extract. There were five replicates in every plate and two plates for each tested extract, for each bacterium. Positive controls consisted of five different antimicrobial susceptibility test discs (Bioanalyse®): furazolidone (100 µg), oxytetracycline (30 µg), tetracycline (30 µg), erythromycin (15 µg) and trimethoprim/sulfamethoxazole (1.25/23.75 µg). Four antibiotic discs were used for each plate and run in duplicate. Water was used as a negative control. Inoculated plates with discs were placed in an incubator at 37 °C, with the exception of *A. salmonicida* and *Y. ruckeri*, which were incubated at 28 °C. After 16 to 18 h of incubation, the inhibition zone diameter (mm) was measured. All experiments were repeated three times.

### Statistical analysis

One-way analysis of variance and Duncan's multiple range tests were run to evaluate the differences between the inhibition zones of the plant extracts, using the SPSS software (Version 15, SPSS Inc., Chicago, IL, USA). The means and standard errors were calculated for each treatment. The accepted level of significance was 0.05.

## Results and discussion

In line with the growing interest in the antibacterial potential of different plants, we examined the antibacterial properties of 72 extracts obtained from 36 plants from the flora of Turkey against seven fish pathogens. The results from the screening study performed by the disc diffusion method are shown in Table 2. The only extracts that did not exhibit any activity against the fish pathogens were the ethanolic extracts of *Eryngium* *campestre* and *Mentha* *longifolia*; the aqueous extracts of *Phlomis* *russeliana*, *Phlomis* *armeniaca*, *Coronilla* *varia*, *Ptilostemon* *afer*, *Daphne* *oleoides*, *Campanula* *glomerata* and *Campanula* *olympica*; and the ethanolic and aqueous extracts of *Viburnum* *opulus*, *Astrantia* *maxima*, *Onobrychis* *oxydonta*, *Astragalus* *brachypterus*, *Doronicum* *orientale*, *Centaurea* *triumfettii*, *Rhinanthus* *angustifolius*, *Cynoglossum* *montanum*, *Echium* *orientale*, *Polygonatum* *orientale*, *Fritillaria* *pontica*, *Arum* *euxinum*, *Hyoscyamus* *niger* and *Asarum* *europaeum*. The largest zone of inhibition was that of the ethanolic and aqueous extracts of *Anemone* *nemorosa*, *Fragaria* *vesca*, *Alchemilla* *mollis* and *Sideritis* *taurica* (endemic) against *V. anguillarum*. The aqueous extracts of *A. nemorosa* showed similar activity to that of erythromycin against *V. anguillarum*. The extracts that showed the broadest antibacterial potential were the ethanolic and aqueous extracts of *Dorycnium* *pentaphyllum* (except for the aqueous extract, which did not show inhibitory activity against *Streptococcus* *agalactiae*). These extracts showed activity against all bacterial strains tested in this study, except *A. salmonicida. V. anguillarum* was inhibited the most, followed by *L. garvieae*, *E. faecalis* and *S. agalactiae*, while no zone of inhibition was observed for *A. salmonicida*. The weakest antibacterial activity was recorded against the Gram-negative bacteria *A. hydrophila* and *Y. ruckeri*. Since the inhibiting activity was higher in ethanolic extracts, alcohol could be considered a better solvent for extraction of antibacterial active substances, compared to water. The largest inhibition zones against bacterial strains were observed in positive controls (reference antibiotics), while there was no inhibition zone in the negative control (water).

**Table 2.** Antibacterial activity of the studied plant extracts*, based on the disc diffusion assay.

		Mean diameter of inhibitory zones (mm ± SE)						
Plants species	Treatment	*A. hydrophila*	*A. salmonicida*	*V. anguillarum*	*Y. ruckeri*	*E. faecalis*	*L. garvieae*	*S. agalactiae*
*C. monogyna*	W	–	–	11.1 ± 0.35 *lmno*	–	–	–	–
	E	–	–	16.6 ± 2.10 *ij*	–	8.3 ± 0.16 *h*	8.8 ± 0.16 *gh*	*–*
*P. coccinea*	W	–	–	10.6 ± 0.18 *mnopr*	–	–	–	–
	E	–	–	12.6 ± 0.42 *l*	9.1 ± 0.23 *g*	9.5 ± 0.19 *f*	10.0 ± 0.27 *hi*	9.1 ± 0.23 *g*
*F. vesca*	W	–	–	19.6 ± 0.18 *g*	–	–	–	–
	E	–	–	19.4 ± 0.26 *g*	–	–	–	–
*R. caesius*	W	–	–	11.5 ± 0.46 *lmn*	–	–	–	–
	E	–	–	10.6 ± 0.42 *mnopr*	–	–	–	–
*A. mollis*	W	16.4 ± 0.46 *e*	–	17.9 ± 0.29 *hi*	10.9 ± 0.29 *f*	*–*	*–*	–
	E	14.0 ± 0.50 *f*	–	–	19.5 ± 0.19 *g*	–	–	–
*V. lantana*	W	–	–	8.6 ± 0.18 *tu*	–	8.5 ± 0.19 *h*	8.3 ± 0.16 *hi*	9.4 ± 0.18 *ij*
	E	–	–	9.8 ± 0.16 *oprst*	–	10.3 ± 0.16 *f*	10.3 ± 0.16 *e*	*–*
*E. campestre*	W	–	–	12.6 ± 0.18 *l*	–	–	–	–
	E	–	–	–	–	–	–	–
*M. longifolia*	W	–	–	15.5 ± 0.33 *jk*	–	–	–	–
	E	–	–	–	–	–	–	–
*L. crinitum*	W	–	–	7.8 ± 0.16 *u*	–	–	–	–
	E	–	–	9.3 ± 0.31 *rsto*	–	–	–	11.3 ± 0.16 *g*
*P. russeliana*	W	–	–	–	–	–	–	–
	E	–	–	9.5 ± 0.19 *prst*	–	–	–	9.3 ± 0.31 *ij*
*P. armeniaca*	W	–	–	–	–	–	–	–
	E	–	–	10.3 ± 0.16 *mnoprs*	–	–	–	–
*S. taurica*	W	10.5 ± 0.46 *g*	–	19.1 ± 0.29 *gh*		–	–	–
	E	–	–	21.5 ± 0.19 *f*	–	–	–	12.3 ± 0.37 *f*
*D. pentaphyllum*	W	7.6 ± 0.18 *h*	–	12.6 ± 0.18 *l*	8.0 ± 0.27 *g*	7.3 ± 0.16 *i*	7.8 ± 0.16 *i*	*–*
	E	10.1 ± 0.58 *g*	–	14.3 ± 0.25 *k*	9.8 ± 0.31 *h*	8.0 ± 0.00 *h*	9.0 ± 0.00 *fg*	9.5 ± 0.19 *ij*
*C. varia*	W	–	–	–	–	–	–	
	E	–	–	11.8 ± 0.67 *lm*	–	–	–	–
*C. triumfettii*	W	–	–	–	–	–	–	–
	E	–	–	7.8 ± 0.16 *u*	–	–	–	–
*P. afer*	W	–	–	–	–	–	10.1 ± 0.29 *e*	
	E	–	–	–	–	–	–	–
*A. nemorosa*	W	–	–	25.5 ± 0.33 *e*	–	–	–	–
	E	–	–	20.5 ± 0.19 *fg*	–	–	–	–
*C. glomerata*	W	–	–	–	–	–	–	–
	E	–	–	11.8 ± 0.16 *lm*	–	–	–	–
*C. olympica*	W	–	–	–	–	–	–	–
	E	–	–	8.6 ± 0.18 *tu*	–	–	–	–
*H. perforatum*	W	–	–	10.9 ± 0.29 *mnop*	–	9 ± 0.27 *g*	9.25 ± 0.16 *fg*	10.6 ± 0.18 *gh*
	E	–	–	9.0 ± 0.10 *stu*	–	7.38 ± 0.18 *i*	8.25 ± 0.16 *hi*	9 ± 0.00 *j*
*H. linarioides*	W	–	–	10.0 ± 0.10 *noprst*	–	7.38 ± 0.18 *i*	8 ± 0.00 *i*	8 ± 0.00 *k*
	E	–	–	10.8 ± 0.16 *mnopr*	–	–	–	–
Negative controls	Water	–	–	–	–	–	–	–
	Ethanol	–	–	–	–	–	–	–
Positive controls	Furazolidone	29.1 ± 0.35 *c*	30.2 ± 0.37 *b*	47.6 ± 0.10 *d*	18.5 ± 0.19 *d*	17.6 ± 0.18 *c*	19.6 ± 0.18 *d*	13.5 ± 0.57 *e*
	Oxytetracycline	32.0 ± 0.76 *b*	26.0 ± 0.38 *c*	51.0 ± 1.51 *c*	33.6 ± 0.18 *c*	11.9 ± 0.12 *e*	33.1 ± 0.35 *b*	36.6 ± 0.18 *d*
	Erythromycin	17.6 ± 0.18 *d*	9.0 ± 0.10 *d*	24.6 ± 0.18 *e*	14.8 ± 0.16 *e*	20.6 ± 0.18 *b*	32.6 ± 0.18 *b*	42.9 ± 0.12 *b*
	Tetracycline	35.8 ± 0.16 *a*	25.9 ± 0.35 *c*	58.3 ± 0.37 *b*	35.8 ± 0.16 *b*	14.5 ± 0.19 *d*	34.8 ± 0.53 *a*	43.9 ± 0.12 *a*
	Trimethoprim /sulfamethoxazole	31.5 ± 0.19 *b*	36.0 ± 0.38 *a*	61.8 ± 0.16 *a*	48.5 ± 0.19 *a*	32.8 ± 0.16 *a*	30.5 ± 0.19 *c*	39.5 ± 0.57 *c*

*Non-active plants are not listed (*Viburnum opulus*, *Astrantia maxima*, *Onobrychis oxydonta*, *Astragalus brachypterus*, *Doronicum orientale*, *Centaurea triumfettii*, *Rhinanthus angustifolius*, *Cynoglossum montanum*, *Echium orientale*, *Polygonatum orientale*, *Fritillaria pontica*, *Arum euxinum*, *Hyoscyamus niger* and *Asarum europaeum*).

W: aqueous extract, E: ethanol extract.

Means with the same letter within columns are not significantly different (*P* > 0.05).

In recent studies, hot ethanolic fruit extracts of *Crataegus* *monogyna*, *Pyracantha* *coccinea*, *V. opulus* and *Viburnum* *lantana* against *Staphylococcus* *aureus*, *Staphylococcus* *epidermidis* and *Streptococcus* *pyogenes* and *Rubus* *caesius* against *S. epidermidis* and *S. pyogenes* showed strong antibacterial activity.[28] Similarly, the antibacterial activity of *V. opulus* and *V. lantana* was expressed against the Gram-negative bacteria, *Escherichia coli* and *Acinetobacter baumanni*.[29] However, *V. lantana* had no inhibitory effect on any of the fish bacteria tested in this study.

The aqueous extract of *F. vesca* leaves has been reported as an antibacterial against *A. hydrophila* and *Y. ruckeri*.[13] In our study, fruits extracts of *F. vesca* in both solvents only inhibited the growth of *V. anguillarum*.

Mkaddem et al. [30] reported that *Listeria monocytogenes* and *Klebsiella pneumoniae* were inhibited by the essential oils of *M. longifolia*. The methanolic extract of *M. longifolia* is also effective against *S.aureus*, *Micrococcus luteus*, *E.coli* and *Pseudomonas aeruginosa*.[31] In our experiment, the aqueous extract of *M. longifolia* showed a strong antibacterial effect only against *V. anguillarum* among the other fish pathogens tested.

The essential oils of *P. russeliana* have been shown to exhibit notable antibacterial activity against common food-borne bacteria, such as *A. hydrophila*, *Bacillus cereus*, *L. monocytogenes*, *P. aeruginosa*, *S. aureus*, *Salmonella typhimurium*, *Yersinia* *enterocolitica* and the anaerobic pathogen *Clostridrium perfringens*.[32] The ethanolic extracts of the same species inhibited the growth of *V. anguillarum* and *S. agalactiae* in our study.

The extracts of *P. armeniaca* in petroleum ether and methanol seem to exert similar antibacterial activity against *S. aureus* and *E. faecalis*.[33] The ethanolic extract of the same species showed inhibitory activity only against *V. anguillarum* in our tests.

While the ethanolic and aqueous extracts obtained from the aerial parts of *H. niger* did not exhibit any antibacterial properties against the fish bacteria tested in this study, the methanolic extract obtained from the seeds of *H. niger* has been shown to exhibit strong antimicrobial properties against *S. aureus*.[34]

There are reports on the antibacterial potential of the alcoholic extract of *Hypericum* species from the Balkans, Pakistan and Turkey. Methanolic extracts of the aerial parts of *H. linarioides* and *H. perforatum* possess a very broad spectrum of strong antimicrobial activity against *S. aureus*, *K. pneumoniae*, *P. aeruginosa*, *Salmonella enteritidis*, *E. coli*, *Aspergillus niger* and *Candida albicans*.[35] Similarly, ethanolic extracts of the aerial parts of *H. perforatum* show considerable activity against *S. aureus* and *P. aeruginosa* [36] and methanolic extracts, against *Klebsiella oxytoca*, *E. coli*, *P. aeruginosa*, *Proteus mirabilis*, *S. aureus*, *L. monocytogenes* and *B. cereus*.[37] In our study, the ethanolic and aqueous extracts from the aerial part of *H. perforatum* were found to be effective against *V. anguillarum*, *L. garvieae, E. faecalis* and *S. agalactiae*. The ethanolic and aqueous extracts of *H. linarioides* were effective against *V. anguillarum* and the ethanolic extract only was effective against *V. anguillarum*, *L. garvieae*, *E. faecalis* and *S. agalactiae*.

To the best of our knowledge, the antibacterial activities of 11 of the plants from Turkey examined by us (*A. mollis*, *Lamium crinitum*, *D. pentaphyllum*, *C.varia*, *D.oleoides*, *A.nemorosa*, *C.glomerata*, *C. olympica*, *S.taurica*, *P.afer* and *Senecio castagneanus*, the last three of which are endemics) have not been hitherto reported.

In the literature, thousands of plant derived compounds have been screened and their inhibitory effects against all types of micro-organisms have been confirmed. With such a growing amount of data, Cowan [15] emphasized that the methods of extraction and *in vitro* testing should be standardized to facilitate the interpretation of the results. After verification and evaluation of *in vivo* bioactivities, isolation and identification of active components from various crude plant extracts should be determined in this kind of screening studies, so that the probability of discovering new drug candidates in aquaculture industry may increase. Further research needs to be focused on subjecting fish to these compounds to determine their effectiveness, stability and impact on the host and on the environment.

## Conclusions

The results from the disc diffusion assay showed that 22 out of 36 plant species from Turkey possess antibacterial activities against pathogenic fish bacteria. *D.pentaphyllum* could be considered a promising source of new drug candidates in aquaculture industry. Further research needs to include *in vivo* tests to determine the effectiveness, stability and impact of the studied extracts (and particular compounds) on fish and on the environment.
